# Supplementary material for: Capsaicin Targets Lipogenesis in HepG2 Cells Through AMPK Activation, AKT Inhibition and PPARs Regulation
Source: Int J Mol Sci. 2019 Apr 3;20(7):1660. doi: 10.3390/ijms20071660 (PMC6480012; doi:10.3390/ijms20071660)
Supplement: Supplementary file 1 [file ijms-20-01660-s001.zip › ijms-470616-supplementary.pptx]

## Slide 1
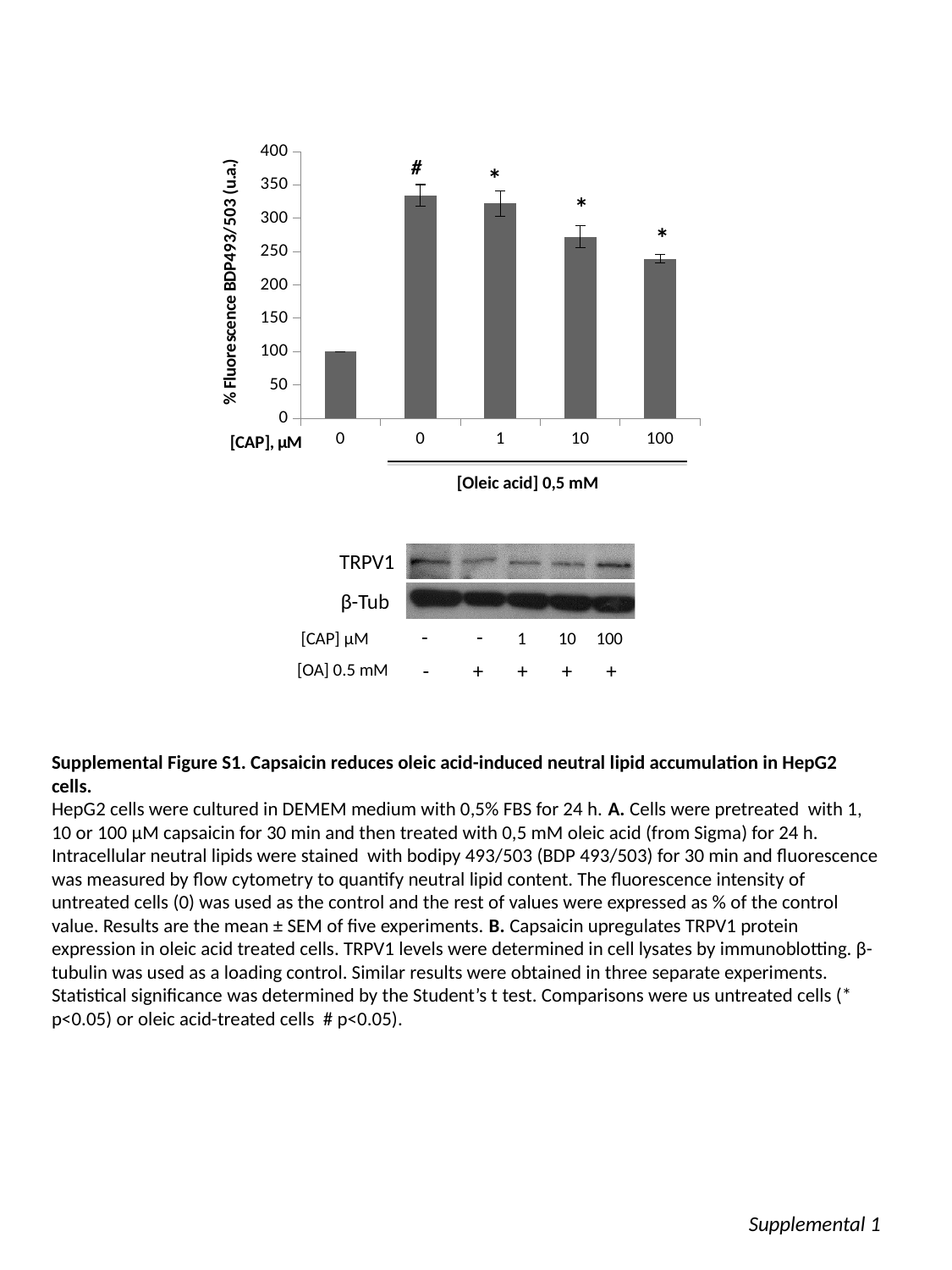

### Chart
| Category | |
|---|---|
| 0 | 100.0 |
| 0 | 334.3072853297653 |
| 1 | 322.3035102009197 |
| 10 | 272.3529322012564 |
| 100 | 239.28975086476765 |[Oleic acid] 0,5 mM
TRPV1
β-Tub
- - 1 10 100
[CAP] µM
- + + + +
[OA] 0.5 mM
Supplemental Figure S1. Capsaicin reduces oleic acid-induced neutral lipid accumulation in HepG2 cells.
HepG2 cells were cultured in DEMEM medium with 0,5% FBS for 24 h. A. Cells were pretreated with 1, 10 or 100 µM capsaicin for 30 min and then treated with 0,5 mM oleic acid (from Sigma) for 24 h. Intracellular neutral lipids were stained with bodipy 493/503 (BDP 493/503) for 30 min and fluorescence was measured by flow cytometry to quantify neutral lipid content. The fluorescence intensity of untreated cells (0) was used as the control and the rest of values were expressed as % of the control value. Results are the mean ± SEM of five experiments. B. Capsaicin upregulates TRPV1 protein expression in oleic acid treated cells. TRPV1 levels were determined in cell lysates by immunoblotting. β-tubulin was used as a loading control. Similar results were obtained in three separate experiments. Statistical significance was determined by the Student’s t test. Comparisons were us untreated cells (* p<0.05) or oleic acid-treated cells # p<0.05).
Supplemental 1

## Slide 2
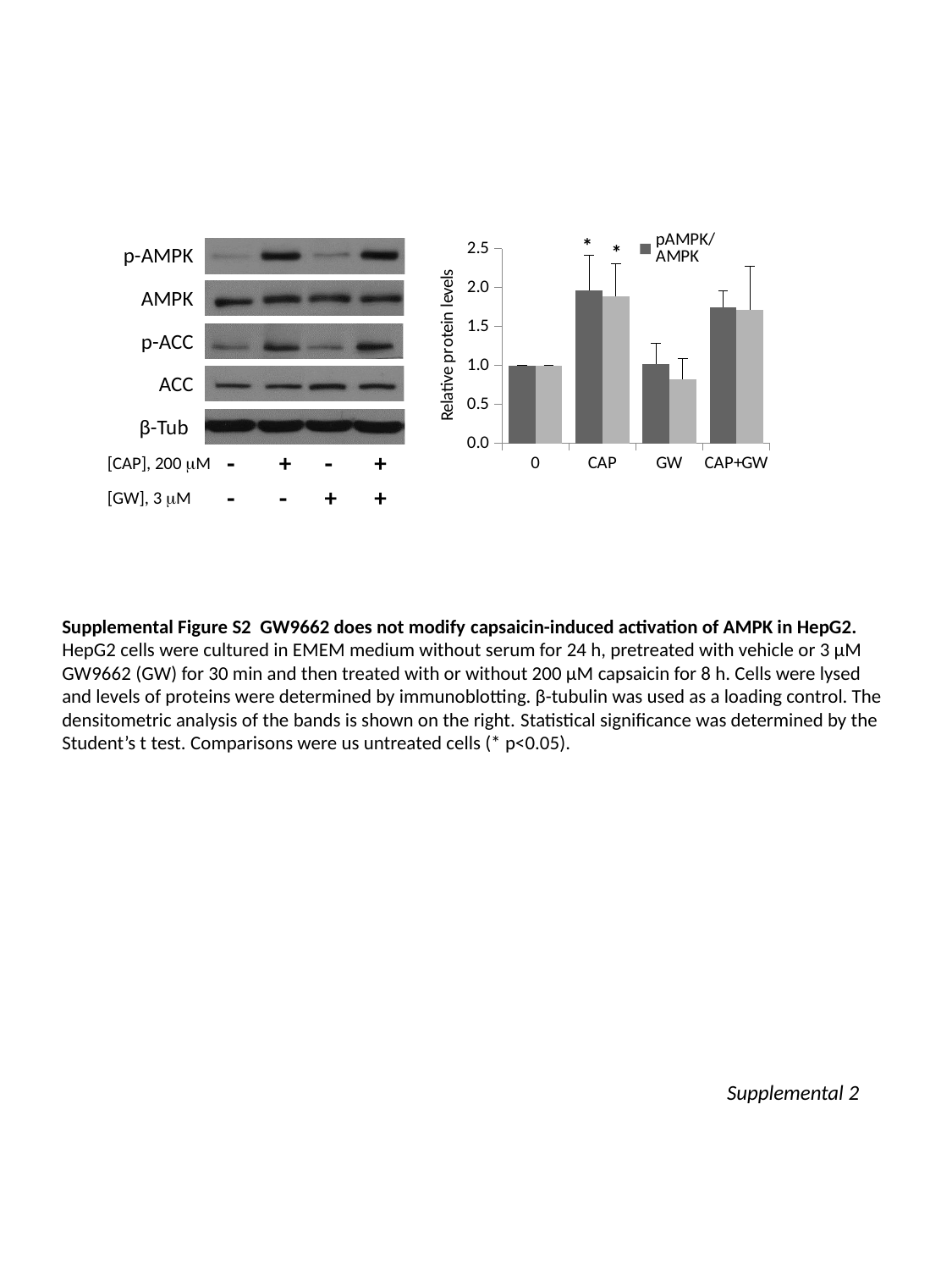

### Chart
| Category | | p-ACC/ACC |
|---|---|---|
| 0 | 1.0 | 1.0 |
| CAP | 1.9655877150011818 | 1.8920152000026296 |
| GW | 1.0192507944379547 | 0.8260557950699563 |
| CAP+GW | 1.7427553448648543 | 1.7173461188324437 |p-AMPK
AMPK
p-ACC
ACC
β-Tub
-
-
+
+
-
-
+
+
[CAP], 200 mM
[GW], 3 mM
Supplemental Figure S2 GW9662 does not modify capsaicin-induced activation of AMPK in HepG2.
HepG2 cells were cultured in EMEM medium without serum for 24 h, pretreated with vehicle or 3 µM GW9662 (GW) for 30 min and then treated with or without 200 µM capsaicin for 8 h. Cells were lysed and levels of proteins were determined by immunoblotting. β-tubulin was used as a loading control. The densitometric analysis of the bands is shown on the right. Statistical significance was determined by the Student’s t test. Comparisons were us untreated cells (* p<0.05).
Supplemental 2

## Slide 3
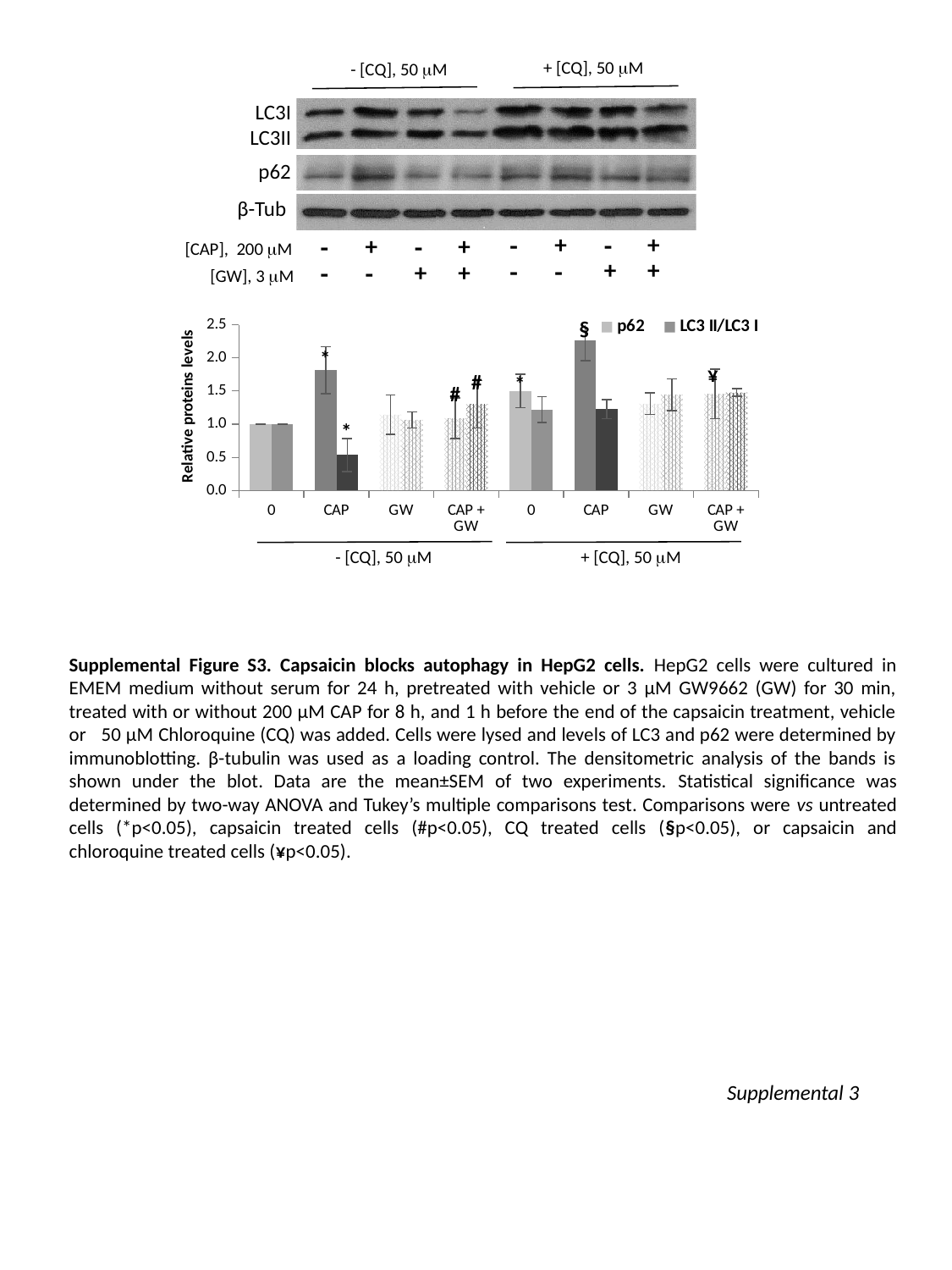

+ [CQ], 50 mM
 - [CQ], 50 mM
LC3I
LC3II
p62
β-Tub
-
+
+
-
-
-
+
+
-
+
+
-
-
-
+
+
[CAP], 200 mM
[GW], 3 mM
### Chart
| Category | p62 | LC3 II/LC3 I |
|---|---|---|
| 0 | 1.0 | 1.0 |
| CAP | 1.8152874937848378 | 0.5345622703078403 |
| GW | 1.1448262569001066 | 1.0666864929262714 |
| CAP + GW | 1.0902289154215354 | 1.3025223995671193 |
| 0 | 1.5002143866048276 | 1.2218953427857346 |
| CAP | 2.267941965100654 | 1.229956193585435 |
| GW | 1.3084476329041754 | 1.445133033008652 |
| CAP + GW | 1.4548129564483532 | 1.4796714704142522 | - [CQ], 50 mM
 + [CQ], 50 mM
Supplemental Figure S3. Capsaicin blocks autophagy in HepG2 cells. HepG2 cells were cultured in EMEM medium without serum for 24 h, pretreated with vehicle or 3 µM GW9662 (GW) for 30 min, treated with or without 200 µM CAP for 8 h, and 1 h before the end of the capsaicin treatment, vehicle or 50 µM Chloroquine (CQ) was added. Cells were lysed and levels of LC3 and p62 were determined by immunoblotting. β-tubulin was used as a loading control. The densitometric analysis of the bands is shown under the blot. Data are the mean±SEM of two experiments. Statistical significance was determined by two-way ANOVA and Tukey’s multiple comparisons test. Comparisons were vs untreated cells (*p<0.05), capsaicin treated cells (#p<0.05), CQ treated cells (§p<0.05), or capsaicin and chloroquine treated cells (¥p<0.05).
Supplemental 3
